# Clinical characteristics and corticosteroids application of different clinical types in patients with corona virus disease 2019

**DOI:** 10.1038/s41598-020-70387-2

**Published:** 2020-08-13

**Authors:** Fangfang Liu, Chengcheng Ji, Jiajun Luo, Weiwei Wu, Junchang Zhang, Zhiqiang Zhong, Seth Lankford, Huang Huang, Fang Lin, Yonggang Wang, Guoxin Mo, Xingshuo Hu, Tianjun Jiang, Yanling Shao, Sumin Ji, Yawei Zhang, Enqiang Qin, Jinsong Mu

**Affiliations:** 1 Fifth Medical Center of PLA General Hospital, Beijing, China; 2 Department of Environmental Health Sciences, Yale School of Public Health, Yale University, New Haven, CT USA; 3 Shanxi Medical University, Taiyuan, Shanxi China; 4 Duke University, Durham, NC USA; 5 First Medical Center of PLA General Hospital, Beijing, China; 6 Department of Surgery, Yale School of Medicine, Yale University, New Haven, CT USA

**Keywords:** Diseases, Infectious diseases, Viral infection

## Abstract

To describe the epidemiological and clinical characteristics of patients with Corona Virus Disease 2019 (COVID-19) in Beijing. To analyze the application of corticosteroids in patients with severe pneumonia. We collected information on demographic characteristics, exposure history, clinical characteristics, corticosteroids use, and outcomes of the 65 confirmed cases of COVID-19 at Fifth Medical Center of PLA General Hospital from Jan 20 to Feb 23, 2020. The final follow-up date observed was April 15th, 2020. The number of patients with mild, general, severe, and critical type were 10 (15.38%), 32 (49.23%), 8 (12.31%), and 15 (23.08%), respectively. The median incubation period was 6 days. Notable outliers were 1 patient at 16 days and 1 patient at 21 days. In lymphocyte subgroup analysis, decreases in total, T, CD4, and CD8 lymphocytes were more common as the disease worsened (All *P* < 0.05). Methylprednisolone (mPSL) was applied to 31 (47.69%) patients with pneumonia, including 10 (31.25%) general, 8 (100%) severe, and 13 (86.67%) critical patients, respectively. Corticosteroids inhibited Interleukin-6(IL-6) production (*P* = 0.0215) but did not affect T lymphocyte (*P* = 0.0796). There was no significant difference between patients using lower dose (≤ 2 mg/kg day) and higher dose (> 2 mg/kg day) mPSL in inhibiting IL-6 production (*P* = 0.5856). Thirty of 31 patients (96.77%) had stopped mPSL due to improvement of pneumonia. Virus RNA clearance time lengthened with disease progression (*P* = 0.0001). In general type, there was no significant difference in virus clearance time between patients with (15, 12–19 days) and without (14.5, 11–18 days) (*P* = 0.7372) mPSL use. Lymphocyte, especially T lymphocyte, in severe and critical patients showed a dramatic decrease. Application of lower dose corticosteroids (≤ 2 mg/kg day) could inhibit IL-6 production (a representative of cytokines) as effectively as a higher dose. Proper use corticosteroids in general type patients did not delay virus clearance.

## Introduction

In December 2019, cases of acute respiratory disease (ARD), now known as a Corona Virus Disease 2019 (COVID-19) occurred in Wuhan, Hubei Province, China^1–3^. Presently, the laboratory-confirmed cases and recorded deaths in the world are still increasing at an alarming rate^4–10^.

COVID-19 clinical types were defined according to the Diagnosis and Treatment of Pneumonia caused by Novel Coronavirus (Version 6 Trial) published on the website of the Central Government of the People’s Republic of China^11^. There are four distinct clinical types based on the severity of the disease. However, the differences in clinical characteristics, corticosteroids application, and outcomes among different clinical types have not been reported.

The pathophysiology of COVID-19 includes severe acute respiratory syndrome coronavirus 2 (SARS-CoV-2) binding to the alveolar epithelium, activating innate immune system and adaptive immune system, resulting in a pro-inflammatory cascade, including the release of Interleukin 6 (IL-6)^12^. Corticosteroid treatment can suppress inflammation, and was used frequently for treatment of patients with severe illness to reduce lung inflammation^13–15^. Corticosteroids were widely used during the outbreaks of severe acute respiratory syndrome (SARS)-CoV and Middle East respiratory syndrome (MERS)-CoV, and are being used in patients with COVID-19^1,16,17^. In light of the urgent clinical demand, physicians from the Chinese Thoracic Society who participated in treating patients with 2019-nCoV pneumonia have developed an expert consensus statement on the use of corticosteroids in COVID-19^18,19^. The outcomes of patients with different COVID-19 clinical types treated by corticosteroids have not been reported.

The aim of the study is to compare clinical characteristics and outcomes among different clinical types of COVID-19 patients treated in a tertiary hospital in Beijing, and to analyze the application of corticosteroids in patients with severe pneumonia.

## Methods

### Data collection

All laboratory-confirmed patients admitted to Fifth Medical Center of PLA General Hospital from January 20 to February 23, 2020, were enrolled. Cases were diagnosed based on Diagnosis and Treatment of Pneumonia caused by Novel Coronavirus (Version 6 Trial) published on the website of the Central Government of the People’s Republic of China^11^. A confirmed case of COVID-19 was defined as a positive result from real-time reverse-transcriptase polymerase-chain-reaction (RT-PCR) assay of respiratory tract specimens^20,21^. Medical records of all confirmed cases with COVID-19 were obtained from electronic medical records. The clinical outcomes, including discharges, mechanical ventilation, intensive care unit (ICU) admission, and death, were recorded up to April 15th, 2020, the final follow-up date for this study.

All medical records were reviewed by a trained team of physicians. Information included demographics, exposure history, concurrent diseases, symptoms, laboratory results, chest X-ray and/or computed tomographic (CT) images, treatment (ie, antiviral therapy, corticosteroids use, respiratory support and continuous renal replacement therapy [CRRT]). Laboratory data included blood routine, biochemistry (ie. liver, kidney and cardiac function, electrolyte, creatine kinase [CK]), coagulation function, infection related biomarkers (ie. C-reactive protein [CRP], procalcitonin [PCT], Interleukin-6 [IL-6], erythrocyte sedimentation rate [ESR], serum ferritin) and lymphocyte subgroup. Acute respiratory distress syndrome (ARDS) was defined according to the Berlin definition^22^. Acute kidney injury (AKI) was identified according to the Kidney Disease: Improving Global Outcomes definition^23^.

Clinical types of COVID-19 included mild, general, severe, and critical types. Mild type was diagnosed as patients with mild clinical symptoms and no pneumonia on radiological imaging. General type was diagnosed as patients with fever, respiratory symptoms, and pneumonia on imaging. Severe type was diagnosed as patients with any one of the following: 1. Respiratory distress, RR (Respiratory rates) ≥ 30 bpm; 2. Peripheral capillary oxygen saturation (SpO_2_) at rest ≤ 93%. 3. Arterial oxygen partial pressure (PaO_2_)/fraction of inspiration O2 (FiO_2_) ≤ 300 mmHg. (1 mmHg = 0.133 kPa). Critical type was diagnosed as patients with any one of the following: 1. Respiratory failure and mechanical ventilation required. 2. Shock. 3. Combined with other organ failures and ICU monitoring and treatment needed^11^.

All methods in this study were performed in accordance with the Diagnosis and Treatment of Pneumonia caused by Novel Coronavirus (Version 6 Trial) published on the website of the Central Government of the People’s Republic of China^11^. This study was approved by the ethics committee of Fifth Medical Center of PLA General Hospital. Written informed consent was waived by the ethics committee of Fifth Medical Center of PLA General Hospital in light of the urgent need to collect clinical data.

### Statistical analysis

Continuous variables were described as mean ± standard deviation (SD) or medians (interquartile range (IQR) values) as appropriate. Categorical variables were summarized as the counts and percentages in each category. Comparisons between different clinical types were conducted using ANOVA for continuous variables when homogeneity of variance is valid, or else using Wilcoxon rank-sum test. Chi-square test was used for categorical variables when homogeneity of variance is valid, or else using Fisher’s exact test. IL-6 values and T lymphocyte counts were tested every 2–3 days. A linear mixed model was built to describe the relationship between clinical type or corticosteroids used and these dynamic IL-6 values or T lymphocyte counts throughout the course of the disease. All analysis used a two-sided *P*-value of 0.05 for statistical significance. Statistical analyses were performed using SAS 9.4 (SAS Institute Inc. Cary. NC. USA).

### Ethical approval and informed consent

This study was approved by the ethics committee of Fifth Medical Center of PLA General Hospital. Written informed consent was waived in light of the urgent need to collect clinical data.

## Results

### Demographic and clinical characteristics

Details of demographic and clinical characteristics are shown in Table 1. A total of 65 patients were included in our study with 10 (15.38%) mild, 32 (49.23%) general, 8 (12.31%) severe, and 15 (23.08%) critical type. The mean (SD) age was 48.4 (18.46) years and 55.38% of them were male. Three patients (4.62%) were under 15 years old (the youngest one was 3 years old), and 15 patients (23.08%) were over 65 years old. All 3 younger patients (all with mild type) had a quick recovery to discharge with median virus RNA clearance time of 4 days. Patients with critical type tended to be older than those with mild type (*P* < 0.0001). There was no significant difference by gender among different clinical types (*P* = 1.000).

**Table 1:** Demographics and baseline characteristics of 65 patients admitted to the 5th Medical Center of PLA General Hospital (Jan 20–Feb 23, 2020) with COVID-19 in different clinical types.

Variables	Total (n = 65)		Mild (n = 10)		General (n = 32)		Severe (n = 8)		Critical (n = 15)		*P*
	N	%	N	%	N	%	N	%	N	%	
**Age, years**											
≤ 65	50	76.92	10	10.00	29	90.63	5	62.50	6	40.00	
> 65	15	23.08	0	0.00	3	9.38	3	37.50	9	60.00	0.0003
**Sex**											
Male	36	55.38	6	60.00	18	56.25	4	50.00	8	53.33	
Female	29	44.62	4	40.00	14	43.75	4	50.00	7	46.67	1
**Exposure history**											
No exposure history	7	10.77	0	0.00	1	3.13	0	0.00	6	40.00	
Recently been to Wuhan	36	55.38	8	80.00	20	62.50	3	37.50	5	33.33	
Contacted with patients with COVID-19	17	26.15	0	0.00	10	31.25	5	62.50	2	13.33	
Contacted with people from Wuhan	5	7.69	2	20.00	1	3.13	0	0.00	2	13.33	0.0006
With at least one underlying disease	22	33.85	1	10.00	8	25.00	4	50.00	9	60.00	0.0264
**Treatment**											
Respiratory support											
Oxygen therapy	14	21.54	0	0.00	14	43.75	0	0.00	0	0.00	
HFNC	8	12.31	0	0.00	0	0.00	8	100.00	0	0.00	
NIPPV	12	18.46	0	0.00	0	0.00	0	0.00	12	80.00	
IPPV	3	4.62	0	0.00	0	0.00	0	0.00	3	20.00	< 0.0001
CRRT	3	4.62	0	0.00	0	0.00	0	0.00	3	20.00	0.0078
Glucocorticoids	31	47.69	0	0.00	10	31.25	8	100.00	13	86.67	< 0.0001
**Prognosis**											
Remained in hospital	0	0.00	0	0.00	0	0.00	0	0.00	0	0.00	
Discharged	63	96.92	10	100.00	32	100.00	8	100.00	13	86.67	
Died	2	3.08	0	0.00	0	0.00	0	0.00	2	13.33	0.124

	Med	IQR	Med	IQR	Med	IQR	Med	IQR	Med	IQR	
Onset of symptom to laboratory confirmed, days	5	3–7	3	1–9	3.5	2.5–6	5.5	2.5–9	7	4–14	0.0004
Onset of symptom to pneumonia, days	6	3–8	4	4–9	5.5	2–7.5	5	2.5–7	7	5–11	0.0443
Onset of symptom to virus RNA clearance, days	15	11–20	6	4–12	14.5	11.5–18.5	18	13–22	20	16–26	0.0001
Methylprednisolone time (n = 31)	7	5–9	–	–	5.5	4–9	7.5	5–10.5	8	6.5–10	0.322
Onset of symptom to discharge, days (n = 63)	27	17–34	13	9–18	24.5	16–32	35	31–44.5	34	28–41	< 0.0001

*COVID-19* Corona Virus Disease 2019, *ICU* intensive care unit, *IQR* interquartile range, *HFNC* high-flow nasal cannula, *NIPPV* non-invasive positive pressure ventilation, *IPPV* invasive positive pressure ventilation, *Med* Median.

A history of recent travel to or living in Wuhan, contact with people confirmed with COVID-19, and contact with people from Wuhan were documented in 55.38%, 26.15% and 7.69% of patients, respectively. Not all patients could provide their exact exposure time. There were 49 patients with complete exposure time information. The range of time from exposure to onset of symptoms (incubation period) was 0 to 21 days, with median (IQR) of 6 (4–10) days. Notably, 2 patients presented at 14 days, 1 patient at 16 days and 1 patient at 21 days.

Twenty-two patients (33.85%) had at least one concurrent disease (i.e. hypertension, diabetes, malignancy, endocrine disease, and tumor). Patients with any concurrent disease were significantly more likely to be diagnosed as critical cases (60% with any concurrent disease) as compared to other patients (mild 10%, general 25%, and severe 50%) (*P* = 0.0264).

Fever (86.15%) and dry cough (56.92%) were the most common symptoms. The mean (SD) maximum temperature was 38.04 (0.86) °C. There was no significant difference in fever and dry cough among patients with different clinical types (all *P* > 0.05). The median (IQR) times of onset of symptom to laboratory confirmation and pneumonia were 5 (3–7) and 6 (3–8) days, respectively. The median (IQR) time of onset of symptom to virus RNA clearance was 15 (11–20) days. Virus RNA clearance time lengthened with disease progression (*P* = 0.0001). The most common pattern on chest X-ray and CT in patients with pneumonia was bilateral patchy shadowing and bilateral ground-glass opacity, respectively.

### Laboratory parameters of patients in different clinical types

**Figure 1:**
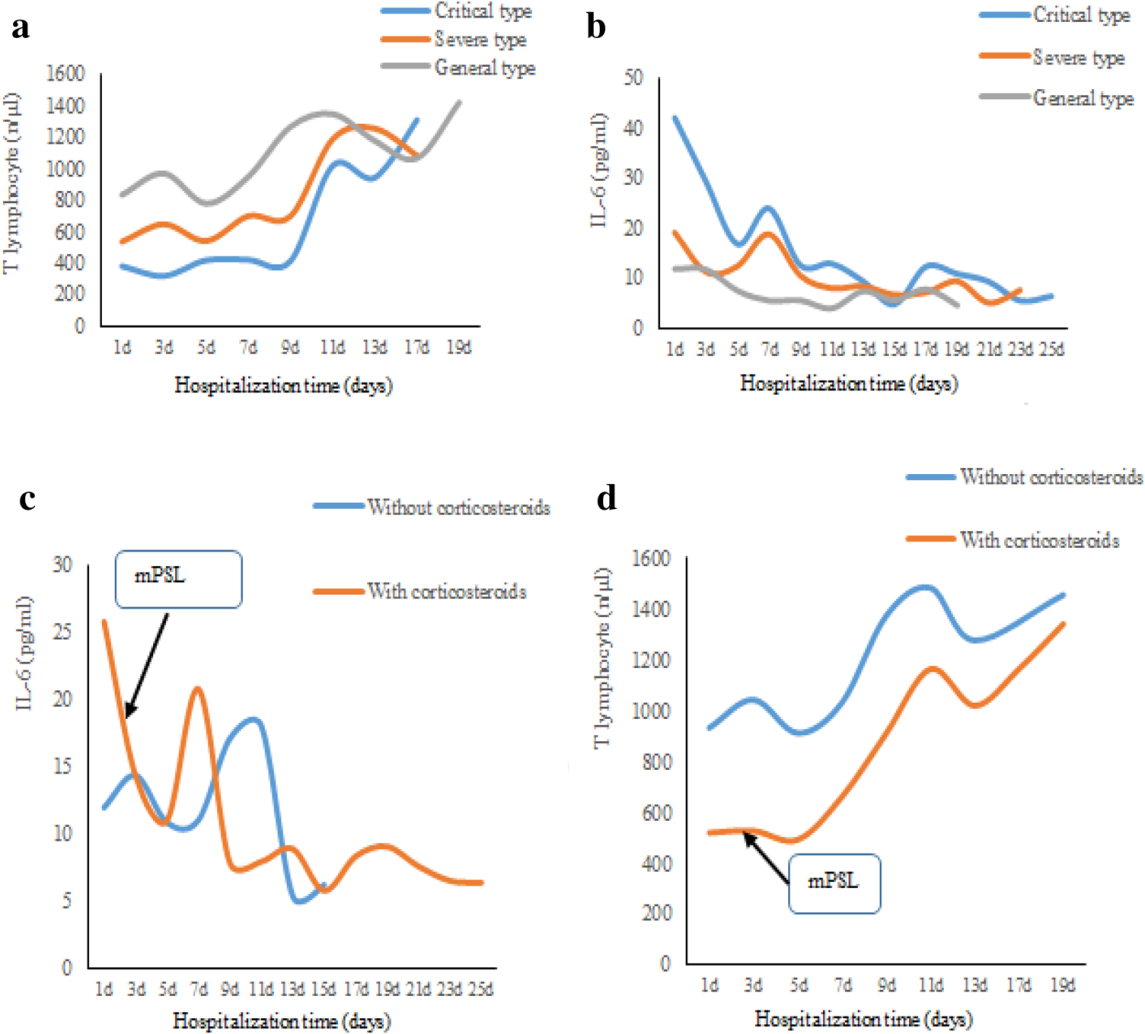
Dynamic IL-6 levels and T lymphocyte counts trends of patients with COVID-19 in different group throughout the course of the COVID-19. (**a**,**b**) showed the T lymphocyte and IL-6 level trends in different clinical types throughout the course of the disease. T lymphocytes increased gradually as patients recovered. During treatment, the IL-6 levels fluctuated and then decreased to normal as patients recovered. There was no records of dynamic T lymphocytes and IL-6 levels in patients with mild type. (**c**,**d**) showed the IL-6 levels and T lymphocyte trends, respectively. The IL-6 levels in patients using corticosteroids decreased quickly after using corticosteroids. Whereas in patients without corticosteroids use, the decrease of IL-6 levels was later and more gradual as patients recovered. The T lymphocyte count trends had a similar pattern throughout the course of disease in patients with and without corticosteroids use. *COVID-19*, Corona Virus Disease 2019, *IL-6* interleukin-6, *mPSL* methylprednisolone.

Details of laboratory results are shown in Table 2, Supplemental Tables S1 and S2. Laboratory abnormalities, including aspartate amino transferase (ALT), CK, potassium, creatine kinase-MB (CKMB), myocardial troponin I (cTnI), myoglobin, N-terminal pro-brain natriuretic peptide (NT-pro BNP), and lactate (LAC), were more common as disease worsened (All *P* < 0.05). Nineteen (29.23%) patients had lymphopenia (lymphocyte count < 1.0 × 10^9^/L). No mild type patients had lymphopenia, however, 73.33% of critical type did (*P* < 0.0001). In lymphocyte subgroup analysis, 20 (30.77%), 29 (44.62%), 35 (53.85%), and 18 (27.69%) patients had decreased total, T, CD4, and CD8 lymphocyte, respectively. Decreases in all four indicators above were more common as the disease worsened (All *P* < 0.05). Meanwhile, lymphocyte counts increased gradually as patients recovered. T lymphocyte count trends in different clinical types throughout the course of the disease are shown in Fig. 1a.

**Table 2:** Infection-related biomarkers and Lymphocyte subgroup of patients with COVID-19 in different clinical types.

	Mild (n = 10)		General (n = 32)		Severe (n = 8)		Critical (n = 15)		*P*
	Mean	SD	Mean	SD	Mean	SD	Mean	SD	
**Infection-related biomarkers**									
IL-6 (pg/ml)	5.36^a^	1.84	13.76^a^	9.07	15.94^a^	14.88	33.21	28.58	0.0003
ESR (mm/h)	8.75^abc^	8.66	29.38^ad^	21.76	33.38^d^	27.32	50.21^cd^	48.00	0.0008
Serum ferritin (ng/ml)	148.22^a^	196.53	396.02	260.29	469.53	188.18	1,005.16^bcd^	577.32	< 0.0001
CRP (mg/l)	7.79^a^	10.88	12.79^ac^	7.70	17.84^a^	15.09	46.75^bd^	37.66	< 0.0001
**Lymphcyte subgroup**									
Total (n/μl)	1,810.57^abc^	730.95	1,266.27^abd^	432.62	902.5^cd^	441.84	512.8^cd^	299.52	< 0.0001
T (n/μl)	1,194.29^abc^	400.10	857.74^abd^	283.59	582.25^acd^	305.80	278.27^bcd^	122.85	< 0.0001
CD4 (n/μl)	685.71^abc^	237.23	454.16^ad^	193.24	317.63^ad^	162.30	139^bcd^	87.86	< 0.0001
CD8 (n/μl)	475.86^ab^	159.60	385.74^ab^	142.82	237.63^cd^	168.54	134.13^cd^	74.90	< 0.0001
B (n/μl)	254.57^ab^	196.68	175.47^a^	100.13	127.88^d^	81.13	97.73^cd^	76.76	0.0133
NK (n/μl)	317.86^a^	245.90	201.23	215.58	165.88	173.58	117	137.44	0.171

*COVID-19* Corona Virus Disease 2019, *IL-6* Interleukin-6, *ESR* erythrocyte sedimentation rate, *CRP*, C-reactive protein.

^a^Compared to critical type, P < 0.05.

^b^Compared to severe type, P < 0.05.

^c^Compared to general type, P < 0.05.

^d^Compared to mild type, P < 0.05.

Forty-four (67.69%), 34 (52.31%), 33 (50.77%), and 35 (53.85%) patients had increased IL-6, ESR, serum ferritin, and CRP, respectively. Increases in all four infection-related biomarkers above were observed more often in critical patients (80%, 80%, 73.33%, and 73.33%, respectively) than in mild patients (10%, 10%, 10%, and 20%, respectively) (All *P* < 0.05). During treatment, the IL-6 levels fluctuated and then decreased to normal as patients recovered. The fluctuation was probably influenced by corticosteroids use. IL-6 trends in different clinical types throughout the course of the disease are shown in Fig. 1b.

### Treatment and clinical outcomes in different clinical types

Details of treatment and clinical outcomes are shown in Table 1. Interferon-α (IFN-α) (59, 90.77%) and lopinavir/ritonavir (50, 76.92%) were the main antivirus medicines used in these patients. Oxygen therapy, high-flow nasal cannula (HFNC), non-invasive positive pressure ventilation (NIPPV), and invasive positive pressure ventilation (IPPV) were applied to 14 (21.54%), 8 (12.31%), 12 (18.46%), and 3 (4.62%) patients, respectively. The median (IQR) time of onset of symptom to HFNC use and mechanical ventilation was 13 (11–19) days and 14.5 (11.5–17) days, respectively. CRRT was applied to 3 critical patients in AKI stage 2.

Methylprednisolone (mPSL) was applied to 31 (47.69%) patients with pneumonia, including 10 (31.25%) general, 8 (100%) severe, and 13 (86.67%) critical patients, respectively. The dosage and duration of mPSL were prescribed individually. In the 31 patients using mPSL, the median (IQR) dosage of mPSL was 1 (1–5) mg/kg day, including 1 (1–2) mg/kg day mPSL in patients with general type, 3 (1–5) mg/kg day mPSL in patients with severe type, and 2 (1–4) mg/kg day mPSL in patients with critical type. We divided patients with mPSL into lower dose (≤ 2 mg/kg day) and higher dose (> 2 mg/kg day) group. The number of patients using lower dose (≤ 2 mg/kg day) and higher dose (> 2 mg/kg day) of mPSL was 20 (64.52%) and 11 (35.48%), respectively.

Thirty of the 31 patients (96.77%) had stopped mPSL due to improvement of pneumonia on chest X-ray/CT or PaO_2_/FiO_2_. One patient died of severe ARDS during mPSL use. Another patient died of septic shock after mPSL use had been stopped 11 days. The median (IQR) time of mPSL use was 7 (5–9) days. The side effects of using mPSL in these 31 patients were hypertension (8, 25.81%), hyperglycemia (11, 35.48%), hypokalemia (11, 35.48%), arrhythmia (4, 12.9%), neuropsychiatric symptoms (2, 6.45%), and gastrointestinal bleeding (1, 3.23%). All above side effects were relived after symptomatic treatment.

The median (IQR) of virus clearance time in patients without mPSL (12.5, 6–17 days) was shorter than in patients with mPSL (19, 14–22 days) (*P* = 0.0003). But in general type, there was no significant difference in virus clearance time between patients with (15, 12–19 days) and without mPSL use (14.5, 11–18 days) (*P* = 0.7372).

Linear mixed models showed the relationship between clinical type and dynamic IL-6 values or T lymphocytes as well as the relationship between corticosteroids use and dynamic IL-6 values or T lymphocytes throughout the course of the disease. When adjusted for age, gender, concurrent disease, and severity of disease (clinical type), corticosteroids inhibited IL-6 levels significantly (*P* = 0.0215) (Table 3) but did not affect T lymphocyte counts (*P* = 0.0796) (Table 5). When adjusted for age, gender, concurrent disease, and corticosteroids use, IL-6 increased dramatically in patients with severe and critical types compared to patients with mild type (All *P* < 0.05) (Table 3), whereas T lymphocytes decreased dramatically in patients with severe and critical types compared to patients with mild type (All *P* < 0.05) (Table 5).

**Table 3:** Linear mixed model for dynamic IL-6 levels with corticorsteroids use (with or without) in patients with COVID-19.

	Estimate	*P*
Without corticosteroids (n = 34)	Ref	
With corticosteroids (n = 31)	− 9.66	0.0215
Mild type (n = 10)	Ref	
General type (n = 32)	7.78	0.1247
Severe type (n = 8)	14.90	0.0385
Critical type (n = 15)	25.35	0.0005
Mild type (n = 10)	− 7.78	0.1247
General type (n = 32)	Ref	
Severe type (n = 8)	7.12	0.1574
Critical type (n = 15)	17.58	0.0006
Mild type (n = 10)	− 14.90	0.0385
General type (n = 32)	− 7.12	0.1574
Severe type (n = 8)	Ref	
Critical type (n = 15)	10.46	0.0195
Mild type (n = 10)	− 25.35	0.0005
General type (n = 32)	− 17.58	0.0006
Severe type (n = 8)	− 10.46	0.0195
Critical type (n = 15)	Ref	

Adjusted for age, gender, cocurrent diseases.

**Table 4:** Linear mixed model for dynamic IL-6 levels with different corticorsteroids dosage (lower and higher dose) in patients with COVID-19.

	Estimate	*P*
≤ 2 mg/kg day (lower dose) (n = 20)	Ref	
> 2 mg/kg day (higher dose) (n = 11)	− 2.33	0.5856

Adjusted for age, gender, cocurrent diseases, clinical type.

**Table 5:** Linear mixed model for dynamic T lymphocytes with corticorsteroids use (with or without) in patients with COVID-19.

	Estimate	*P*
Without corticosteroids (n = 34)	Ref	
With corticosteroids (n = 31)	− 190.82	0.0796
Mild type (n = 10)	Ref	
General type (n = 32)	− 310.12	0.06
Severe type (n = 8)	− 450.45	0.0343
Critical type (n = 15)	− 672.74	0.0009
Mild type (n = 10)	310.12	0.06
General type (n = 32)	Ref	
Severe type (n = 8)	− 140.33	0.3011
Critical type (n = 15)	− 362.63	0.0025
Mild type (n = 10)	450.45	0.0343
General type (n = 32)	140.33	0.3011
Severe type (n = 8)	Ref	
Critical type (n = 15)	− 222.30	0.0982
Mild type (n = 10)	672.74	0.0009
General type (n = 32)	362.63	0.0025
Severe type (n = 8)	222.30	0.0982
Critical type (n = 15)	Ref	

Adjusted for age, gender, cocurrent diseases.

Another two linear mixed models showed the relationship between corticosteroids dosage (lower and higher dosage) and dynamic IL-6 values or T lymphocytes in patients applied with mPSL. When adjusted for age, gender, concurrent disease, and severity of disease (clinical type), there was no significant difference between patients using corticosteroids at lower (≤ 2 mg/kg day) and higher dose (> 2 mg/kg day) (All *P* > 0.05) (Tables 4, 6).

**Table 6:** Linear mixed model for dynamic T lymphocytes with different corticorsteroids dosage (lower and higher dose) in patients with COVID-19.

	Estimate	*P*
≤ 2 mg/kg day (Lower dose) (n = 20)	Ref	
> 2 mg/kg day (Higher dose) (n = 11)	136.56	0.244

Adjusted for age, gender, cocurrent diseases, clinical type.

The IL-6 levels in patients using corticosteroids decreased quickly after using corticosteroids. Whereas in patients without corticosteroids use, the decrease of IL-6 levels was later and more gradual as patients recovered (Fig. 1c). The T lymphocyte count trends had a similar pattern throughout the course of disease in patients with and without corticosteroids use (Fig. 1d).

The number of patients admitted to the ICU and deceased were 6 (9.2%) and 2(3.08%), respectively. The time from onset of symptoms to death of the 2 deceased patients were 13 and 27 days, respectively. 63 patients were discharged with full recovery. In these patients, the median (IQR) time from onset of symptoms to discharge was 27(14–34) days, including 13 (9–18) days for mild, 24.5 (16–32) days for general, 35(31–44.5) days for severe, and 34 (28–41) days for critical patients (*P* < 0.0001).

## Discussion

Our study included 65 COVID-19 patients representing the full spectrum of clinical types. All patients, except for 16 who had no identified exposure history, were infected through human-to-human transmission. Human-to-human transmission was the major way spreading COVID-19 in our study, which is consistent with recent reports^5–7,9^. The median incubation period was 6 days, including 2 patients at 14 days, 1 patient at 16 days and 1 patient at 21 days. 14 days may not be long enough to rule out infection after coming in contact with infected patients. The incubation period needs to be verified with a larger population.

Fever, dry cough and bilateral patchy shadowing on chest X-ray were the most common clinical features, whereas other symptoms (ie. myalgia, fatigue, dyspnea, anorexia, diarrhea, nausea and vomiting) were observed in less than 35% of cases. Chest CT feature was more typical with bilateral ground-glass opacity. The duration of time from onset of symptoms to pneumonia was similar in different clinical types. However, the onset of symptom to virus RNA clearance time lengthened dramatically as clinical type worsened.

The laboratory abnormalities observed in this study were leucopenia, lymphopenia, elevated D-dimer, ESR, IL-6, serum ferritin, CRP, and LAC, which were related to sustained inflammatory response. All the observed abnormalities were more pronounced in critical patients than in mild ones. We also observed that COVID-19 is more severe in older people as well as patients with concurrent diseases as these patients generally had weaker immune functions^24,25^. Decreased T, CD4, and CD8 lymphocytes were much more common in critical patients than in mild ones in our study. A substantial decrease in total number of lymphocytes indicates that coronavirus consumes many immune cells and inhibits the body’s cellular immune function. Damage to T lymphocytes might be an important factor leading to exacerbations of patients^26^. All the evidence indicates that COVID-19 might mainly act on lymphocytes, especially T lymphocytes. In this study, decreased lymphocytes, including total, T, CD4, and CD8 lymphocytes, were more common as the disease worsened.

The SARS-CoV-2 could activate the immune system and induce a pro-inflammatory cascade^12^. IL-6 plays an important role in COVID-19-induced cytokine storm, characterized by an extreme auto-amplifying cytokine reaction which is followed by infiltration of inflammatory monocytes/macrophages and lymphocytes into the lung^27^. In treatment of SARS-CoV^13,14^ and MERS-CoV^15^, corticosteroids were commonly used to treat patients with severe illness by reducing lung inflammatory. Some reports did not support corticosteroid treatment, because it could inhibit immune responses and virus clearance^28–30^. However, some studies supported the use of corticosteroids in patients with coronavirus infection because it could reduce mortality and the need for mechanical ventilation, and shorten the length of stay in hospital^31–33^. In COVID-19 treatment, the mPSL was applied according to the Chinese COVID-19 corticosteroids use recommendation^11,18^, severity of pneumonia, the progress of inflammation, and the patients’ response to mPSL. Therefore the dosage and duration were individually. In this study, mPSL was applied to 47.69% of patients with COVID-19, including 31.25% general, 100% severe, and 86.67% critical patients, respectively. Thirty of the 31 patients (96.77%) with mPSL using had stopped mPSL due to improvement of pneumonia. We found that corticosteroids inhibited IL-6 production throughout the course of the disease. Meanwhile, there was no significant difference between patients using lower dose (≤ 2 mg/kg day) and higher dose (> 2 mg/kg day) mPSL. Therefore, application of lower dose corticosteroids (≤ 2 mg/kg day) could inhibit IL-6 production (a representative of cytokines) as effectively as a higher dose.

Some studies reported that mPSL may delay virus clearance time^17,34^. In our study, all severe and most critical patients used mPSL. We could not distinguish whether the lengthened virus RNA clearance time was completely due to disease progression or if it was impacted by mPSL use. We could not compare the virus clearance time of using versus not using mPSL in severe and critical groups due to almost all of these patients being treated with mPSL. In general type, 31.25% of patients used mPSL and 68.75% of patients didn’t use, and the virus clearance time was similar between users and non-users. Therefore, more study is necessary to elucidate whether properly using corticosteroids delays virus clearance time among patients with COVID-19.

Timely use of mechanical ventilation is a more effective treatment for patients with moderate or severe ARDS^35^. NIPPV or IPPV could open alveoli and reduce exudation, improving the PaO_2_/FiO_2_, which is the signal to stop using mPSL. At the same time, preventing fluid overload and early application (AKI stage 2) of CRRT also guarantee a better prognosis.

Proper use of corticosteroids could have led to the improvement observed in this study. The following rules should be considered when using corticosteroids: 1. When to start and stop using corticosteroids should be carefully weighed. 2. Dosage should be personalized, and the duration should be short, especially in critical patients. 3. Side effects should be seriously monitored and promptly addressed.

IL-6 plays an important role in COVID-19 induced cytokine storm^27^. Elevated levels of IL-6 are predictive of a fatal outcome in COVID-19^12^. In order to decrease the cytokine storm, aside from corticosteroids, Tocilizumab (TCZ) has been proposed to treat severe forms of COVID-19^27^. TCZ is a humanized recombinant monoclonal antibody that acts as an IL-6 receptor antagonist, specifically binding to soluble or membrane-type IL-6 receptors. Several studies have shown very good effects of tocilizumab in patients with COVID-19^36,37^. Meanwhile, some studies also found its rare but possible serious hepatotoxicity, especially when used together with other hepatotoxic drugs^37^.

This study has several limitations. Only 65 patients with confirmed COVID-19 in Beijing were included, thus certain subgroup analyses had limited statistical power, such as treatment effect comparison and mortality. We will continue to collect multi-center cases in order to have a more comprehensive understanding of COVID-19.

## Conclusion

The incubation period of COVID-19 in a few patients is likely to be greater than 14 days. Lymphocyte, especially T lymphocyte, in severe and critical patients showed a dramatic decrease. Application of lower dose corticosteroids (≤ 2 mg/kg day) could inhibit IL-6 production (a representative of cytokines) as effectively as a higher dose. Proper use of corticosteroids in general type patients did not delay virus clearance. For patients with moderate to severe ARDS, timely use of mechanical ventilation and CRRT guarantees a better prognosis.

## Supplementary information

Supplementary Table S1.

Supplementary Table S2.
